# *Pi∗S* and *Pi∗Z* Alleles of *SERPINA1* Gene Are Associated With Specific Variants of a BRD4-Independent Enhancer

**DOI:** 10.1155/2024/6472805

**Published:** 2024-06-25

**Authors:** Ainhoa Escuela-Escobar, Esther Herrera-Luis, Elena Martín-González, José María Hernández-Pérez, Mario A. González Carracedo, José Antonio Pérez Pérez

**Affiliations:** ^1^ Genomics and Health Group Department of Biochemistry Microbiology Cell Biology and Genetics Universidad de La Laguna (ULL), 38200 La Laguna, Tenerife, Spain; ^2^ Genetics Laboratory Institute of Tropical Diseases and Public Health of the Canary Islands (IUETSPC) Universidad de La Laguna (ULL), 38200 La Laguna, Tenerife, Spain; ^3^ Department of Epidemiology Bloomberg School of Public Health Johns Hopkins University, Baltimore, Maryland, USA; ^4^ Department of Respiratory Medicine Hospital Universitario de N. S de Candelaria (HUNSC), 38010 Santa Cruz de Tenerife, Tenerife, Spain

**Keywords:** Alpha-1 antitrypsin, BRD4-Independent Enhancer, CpG, *Pi∗S*, *Pi∗Z*, *SERPINA1*

## Abstract

Alpha-1 antitrypsin deficiency (AATD) is a genetic disorder caused by specific variants in the *SERPINA1* gene, which encodes AAT. The most common disease-associated *SERPINA1* variants are *Pi*∗*S* and *Pi*∗*Z* alleles, which cause moderate and severe AATD, respectively. Recent studies have reported the presence of a possible regulator of *SERPINA* gene cluster expression (LOC126862032), which is suggested to act as a BRD4-Independent Enhancer (*SERPINA*-BIE). This study is aimed at characterizing the *SERPINA*-BIE locus and assessing possible associations with *SERPINA1* AATD-related alleles. For this purpose, *SERPINA*-BIE was PCR genotyped from 917 samples, including 452 asthmatic patients, and 465 newborns. Nine *SERPINA*-BIE alleles were sequenced, revealing a specific combination of 56-bp sequence types, and each *SERPINA*-BIE allele has a unique total number of CpG sites. Statistical analyses revealed an association between the *Pi*∗*Z* allele of the *SERPINA1* gene and the *SERPINA*-BIE allele 13 (*p* value = 5.51 × 10^−10^), as well as between *Pi*∗*S* and *SERPINA*-BIE allele 14 (*p* value = 8.95 × 10^−15^). However, AAT levels were not associated with *SERPINA*-BIE alleles when models were corrected by *SERPINA1* genotypes. This study could contribute to a better understanding of the regulation of the *SERPINA1* gene expression, and its role in AATD.

## 1. Introduction

*SERPINA1* gene encodes alpha-1 antitrypsin (AAT) in humans, a serum monomeric glycoprotein of 52 kDa. This gene is mapped on the minus DNA strand, in the chromosomal region 14q31-32.3, spanning 13,889 nt within the *SERPINA* gene cluster. *SERPINA1* gene contains six introns and seven exons, which are divided into three noncoding exons (Ia, Ib, and Ic), and four coding exons (II, III, IV, and V). Different promoters and transcription initiation sites have been identified for macrophages and hepatocytes, revealing an extraordinary complex regulation of *SERPINA1* gene expression [1]. AAT is the most important antiprotease in serum, protecting several tissues against the elastin degradation mediated by the neutrophilic elastase, especially the lungs [2]. AAT is predominantly synthesized in the liver and subsequently released into the bloodstream. The expression of the *SERPINA1* gene is highly regulated at different levels. Specifically, the upregulation of the *SERPINA1* expression is necessary to control elastase activity during infections or immune response, acting as an acute phase reactant [3]. In addition to its antiprotease activity, different immunomodulatory properties have been more recently attributed to AAT, thus suggesting an important role in the modulation of the inflammatory response [4].

One of the most frequent conditions among rare genetic disorders worldwide is AAT deficiency (D), which affects about one in 2000 to one in 5000 Caucasian individuals [5]. Classically, isoelectric focusing (IEF) electrophoresis has been used to identify different AAT isoforms present in serum [6]. The most common phenotypes are known as *Pi*∗*M*, *Pi*∗*S*, or *Pi*∗*Z*, where *Pi*∗*M* represents the AAT isoforms with the reference migration pattern, while *Pi*∗*S* and *Pi*∗*Z* show more cathodic positions in the IEF gel [7]. The *Pi*∗*M1-val213* allele is considered the reference *SERPINA1* sequence, and it is associated with normal serum AAT levels. In contrast, AATD is characterized by a reduction in AAT serum levels or activity, and in 98% of cases, it is caused by two different variants of *SERPINA1* gene [8], the so-called *Pi*∗*S* (T-allele of rs17580, in exon III), and *Pi*∗*Z* alleles (A-allele of rs28929474, in exon V) [6]. Moreover, many other rare mutations have been also associated with this condition [9].

AATD can lead to lung and liver clinical manifestations. Lung diseases mainly encompass chronic obstructive pulmonary disease (COPD) and panacinar emphysema, while liver diseases can manifest as neonatal cholestasis, juvenile hepatitis, liver cirrhosis in children and adults, and hepatocellular carcinoma [10]. A decrease in serum AAT levels below a proposed protective threshold (57 mg/dl) [11] increases the risk of lung emphysema and COPD, especially in smokers, since balanced AAT levels are necessary to protect the lung alveoli from elastin degradation caused by neutrophil elastase [10]. Liver disease is frequently associated with *Pi*∗*Z*, as this AAT isoform can form polymers that are retained in the endoplasmic reticulum of hepatocytes, causing endoplasmic reticulum stress, inflammation, and liver fibrosis, which can progress to cirrhosis or hepatocellular carcinoma [12].

Interestingly, a wide range of AAT levels has been observed within each *Pi*∗*MM*, *Pi*∗*MS*, and *Pi*∗*MZ* genotypes. Serum AAT levels for the *Pi*∗*MM* genotype are usually in the range of 103–200 mg/dl. However, in *Pi*∗*MS* individuals, the AAT level ranges between 100 and 180 mg/dl, and for *Pi*∗*MZ* in the 66–120 mg/dl interval [13]. Moreover, *SERPINA1* gene expression is highly induced during the inflammatory response, and AAT levels can be increased three- to fourfold during these episodes [14].

Several studies have examined the mechanisms underlying the regulation of *SERPINA1* gene expression in different cell types, and during the development of various diseases. It has been shown that alternative splicing of the *SERPINA1* mRNA generates tissue-specific isoforms, which can be influenced by various contextual factors [1]. In the liver, the expression of *SERPINA1* is regulated by both transcriptional and posttranscriptional processes. Transcription factors, such as C/EBP*β*, C/EBP*α*, and HNF-1*α*, have been reported to bind the hepatocyte promoter region of the *SERPINA1* gene, inducing its expression [15]. Additionally, miRNAs, specially miR-320c, can regulate *SERPINA1* expression by targeting the 3′UTR region of *SERPINA1* mRNA [16]. Moreover, in lung macrophages, the transcription factor *Nuclear Factor kappa B* (NF-*κ*B) has been found to regulate *SERPINA1* expression [17], which is increased in response to oxidative stress, a common feature of COPD [18].

Previous studies have also shown an association between AATD and other inflammatory diseases [19], including atopy [20], panniculitis [21], vasculitis [22], and asthma [23, 24]. Environmental and genetic factors play crucial roles in the development of these complex diseases, and the mechanisms involved in their interplay are not completely known. However, changes in DNA methylation of specific CpG sites have been proposed as a possible mechanism that underlies this connection [25]. Indeed, DNA methylation has been shown to regulate the expression of the *SERPINA1* gene, and differential methylation of a specific CpG site has been associated with lung function in adult smokers [26, 27]. Moreover, exposure to environmental factors, such as cigarette smoke, has been associated with decreased *SERPINA1* gene expression in lung tissue [28]. A recent study has identified a CpG site in a 1200-bp region (LOC126862032) [29], mapped 44.7 kb downstream of *SERPINA1* gene exon Ia. Differential methylation of this CpG (cg08257009) has been associated with the forced expiratory volume in 1 s/forced vital capacity (FEV_1_/FVC) ratio in adults, thus suggesting a regulatory role over *SERPINA1* gene expression [26]. The regulatory potential of this region was confirmed in a colorectal carcinoma cell line using the STARR-seq massively parallel reporter assay [29]. Therefore, this locus could act as a regulator of the *SERPINA* gene cluster expression, but its regulatory activity over the *SERPINA1* gene has not been experimentally confirmed in hepatocytes, which is the most relevant cell type involved in AAT synthesis. Specifically, LOC126862032 is dependent on BRD2, P300/CBP, MED14, and CDK7 cofactors, while having limited or no reliance on the BRD4 bromodomain protein [29]. Therefore, we have called this locus as *SERPINA* BRD4-Independent Enhancer (*SERPINA*-BIE). In the present work, we have characterized the molecular structure of *SERPINA*-BIE locus for the first time, and the associations between *SERPINA*-BIE alleles and their CpG content, both with AAT levels and *SERPINA1* deficient alleles, have been evaluated.

## 2. Methods

### 2.1. Study Design and DNA Extraction

Dried blood spot samples were collected from 452 asthmatic patients (64.6% female, mean age (interquartile range): 47.1 (32.0-63.0)) and 465 newborns (46.8% female) during 2014 [30]. These individuals were recruited at the allergology, pulmonology, or pediatric services of the Hospital General de La Palma (HGLP), Canary Islands (Spain). During recruitment, AAT protein levels were measured in fresh blood samples by immune nephelometry, using standardized laboratory procedures. Demographic and clinical data were obtained through questionnaires that included variables of interest such as age, sex, BMI, pre-FVE_1_, pre-FVC, exacerbations, asthma control, AAT levels, *SERPINA1* genotypes (*Pi*∗*MM*, *Pi*∗*MS*, *Pi*∗*MZ*, *Pi*∗*SS*, *Pi*∗*SZ*, and *Pi*∗*ZZ*), immunoglobulin E (IgE) level, and eosinophil count, among others. However, clinical data were not available for newborns, except biological sex, and *SERPINA1* genotypes.

Alkaline extracts were prepared from each sample, as explained elsewhere [31]. Extracts were stored at −20°C until use, and working dilutions were prepared by mixing 50 *μ*l of alkaline extracts with 25 *μ*l 10 mM Tris pH 8.0 and also stored at −20°C.

### 2.2. PCR Genotyping of *SERPINA*-BIE Locus

Oligonucleotides for PCR amplification of *SERPINA*-BIE locus were designed with GeneRunner v6.5.52 software [32] (Table 1). PCR reactions were prepared in 96-well plates. Each PCR contains 5 *μ*l of a 2.5-fold dilution of DNA extract, 4 *μ*l of 5X Phire Reaction Buffer (Thermo Scientific, USA), 2 *μ*l of dNTPs (2 mM each), 2 *μ*l of each primer (2 *μ*M), and 0.2 *μ*l of Phire Hot Start II DNA Polymerase (Thermo Scientific, USA). For negative controls, 5 *μ*l of H_2_O was added, instead of template DNA. The final volume was adjusted to 20 *μ*l with H_2_O. A ProFlex PCR System (Thermo Scientific, USA) was used, including an initial denaturation step (98°C; 30 s), followed by 35 cycles of denaturation (98°C; 10 s), annealing (60°C; 10 s), and extension (72°C; 30 s). A final extension step was also included (72°C; 120 s).

Gel electrophoresis was carried out using 1.5% agarose and prepared in 1X TBE buffer and incorporating High-Range DNA Ladder (AppliChem, Germany) as molecular weight reference. Electrophoresis was performed for 2 h at 190 V. For visualization, gels were submerged in 1X GelRed solution (BIOTIUM, USA), for 30 min, and images were captured under ultraviolet light.

For the identification of each *SERPINA* BIE allele, at least two independent interpretations were carried out. The expected length for the amplicon from the *SERPINA*-BIE reference allele was 693 bp, according to GRCh38 reference genome, which consists of 11 repetitions of a 56-bp region (allele 11). Therefore, each *SERPINA*-BIE allele was called considering the number of 56-bp repeats, according to the length estimated by electrophoresis.

### 2.3. Sequencing and CpG Calling

A total of 22 different homozygous individuals were selected for sequencing of alleles 9, 10, 11, 14, 15, and 16. In the cases of alleles 8, 12, and 13, as no homozygous individuals were detected after genotyping, PCR products from eight heterozygous individuals that contain these alleles were selected and cloned in a plasmid vector using the CloneJET PCR Cloning Kit (Thermo Scientific, USA). Briefly, eight PCR products that contain each allele were mixed and purified using magnetic beads (AMPure XP Bead-Based Reagent). Purified amplicons were quantified using a DeNovix spectrophotometer (DeNovix Inc., USA), and 25 ng was mixed with 50 ng of pJET1.2/blunt Cloning Vector (Thermo Scientific, USA). Competent *E. coli* TOP10 cells were transformed by the heat-shock method, as described elsewhere [33]. After 24 h of incubation at 37°C in LB plates supplemented with 10 ng/*μ*l ampicillin, eight transformant colonies were selected with sterile toothpicks and suspended in 200 *μ*l of H_2_O for checking. Colony-PCR reactions include 2 *μ*l of bacterial suspension, 4 *μ*l of 5X Phire Reaction Buffer (Thermo Scientific, USA), 2 *μ*l of dNTPs (2 mM each), 2 *μ*l of each primer (2 *μ*M), 2 *μ*l of BSA (5 *μ*g/*μ*l), and 0.2 *μ*l of Phire Hot Start II DNA Polymerase (Thermo Scientific, USA). PCR volume was adjusted to 20 *μ*l with H_2_O. For negative control reactions, 2 *μ*l of H_2_O was added, instead of bacterial suspension. Amplification conditions were exactly the same as described for genotyping, but including 25 PCR cycles. Electrophoresis was carried as described above. DNA fragments with the expected length for alleles 8, 12, or 13 were selected for Sanger's sequencing.

PCR products were enzymatically cleaned using ExoCleanUp FAST (VWR, USA), following the manufacturer's instructions, and 5 *μ*l was mixed with the same volume of the sequencing primer (5 *μ*M) (Table 1). Samples were delivered to Macrogen INC (South Korea) for Sanger's sequencing. Sequences were inspected and aligned using MEGA v.11.0 software [34], to confirm the 56-bp repetition pattern, and the number of CpG sites present in each specific allele.

### 2.4. Statistics Analysis

Data analysis was performed using RStudio v4.2.3 [35]. Descriptive statistics were obtained for each variable through the *describe* function, and interquartile ranges using the *quantile* function. The predicted percentage of FEV_1_ (pre-FEV_1_) and FVC (pre-FVC) was obtained with the *rspiro* package [36]. Asthma control was assessed considering the Asthma Control Test (ACT). Uncontrolled asthma was defined when ACT < 20 [37]. Exacerbations were defined by requiring corticosteroid use, emergency room visit, and/or hospitalizations, in the past year [38]. For IgE levels, eosinophil counts, and AAT serum levels, outliers were previously visualized and removed using the *boxplot$out* function.

To compare descriptive statistics between asthmatic patients and newborns, each variable was tested for normal distribution using either the Kolmogorov–Smirnov test (*ks.test*) or the Shapiro–Wilk test (*shapiro.test*), when the sample size was higher or lower than *n* = 50, respectively. The Mann–Whitney *U*-test (*wilcox.test*) was applied to compare independent variables without normal distribution, while Student's *t*-test (*t.test*) was applied when normality was found. Hardy–Weinberg equilibrium (HWE) was tested for both populations using the *hwe* function of the *gap* package [39]. Statistical significance was declared based on a 95% confidence interval (95% CI) (*p* value < 0.05).

An ANOVA study was conducted to compare AAT levels between the homozygous individuals. Data normality was verified by the *ks.test*, and Levene's test (*leveneTest*) was used to confirm the homoscedastic distribution of the data. ANOVA was applied using the *aov* function, and the differences between groups were analyzed with the Tukey test (*TukeyHSD*). However, when the variable did not fit the normality distribution or/and homoscedastic distribution, the Kruskal–Wallis test (*kruskal.test*) was used. Statistical significance was declared based on 95% CI (*p* value < 0.05).

Multiple linear regression models (*lm*) were used to investigate the associations between AAT levels, and the presence of 0, 1, or 2 copies of each *SERPINA*-BIE allele. Moreover, the number of CpG sites per allele (CpG_N_), in which *SERPINA*-BIE alleles were joined to define two groups (CpG_N_ ≤ 30 or GpG_N_ ≥ 38), was alternatively used in the regression models. Covariates used for model adjustment were sex, age, *SERPINA1* genotypes (*Pi*∗*MM (non-S/non-S; non-Z/non-Z)*, *Pi*∗*MS (non-S/S; non-Z/non-Z)*, or *Pi*∗*MZ (non-S/non-S; non-Z/Z)*), and/or principal components (PCs) derived from genomic-level genotyping data [23].

To assess the association between *SERPINA1 Pi*∗*MM*, *Pi*∗*MS*, and *Pi*∗*MZ* genotypes with *SERPINA*-BIE alleles, or their CpG_N_, multiple logistic regression models (*lm*, family =  ^“^binomial^”^) were performed, adjusted by sex, age, and/or PCs. Subjects with *Pi*∗*MM* genotype were compared with individuals with *Pi*∗*MS* and *Pi*∗*MZ* genotypes, independently. The odds ratio (OR) value was calculated with the expression OR = EXP (*β*), and the 95% CI was calculated according to the expression 95%CI = EXP (*β* ± (1.96 × *β*_Standard error_)). The final regression models were selected based on the lowest significance value, and statistical significance was declared based on 95% CI (*p* value < 0.05).

## 3. Results

### 3.1. *SERPINA*-BIE Locus Shows a Complex Repetition Pattern, With a Specific CpG Number for Each Allele

**Figure 1:**
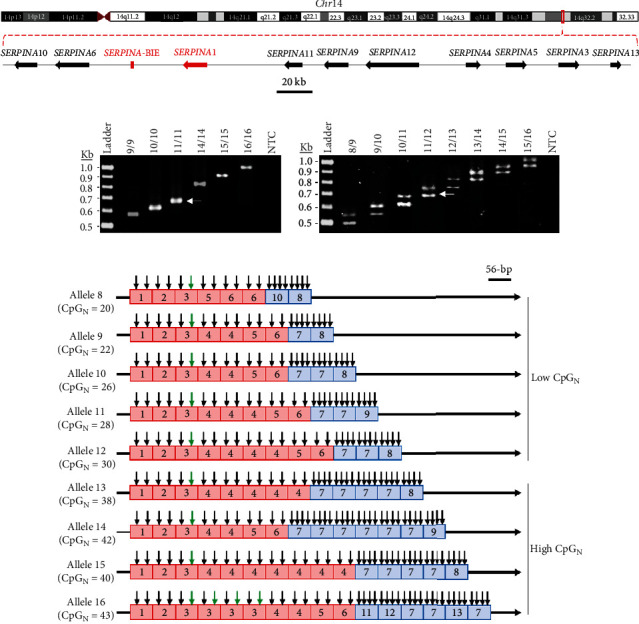
Molecular characterization of *SERPINA*-BIE locus. (a) Genomic structure of *SERPINA* gene cluster at the14q32.1 chromosomal region. The *SERPINA1* gene and *SERPINA*-BIE locus (LOC126862032) are shown in red. (b) Example of genotyping results, from homozygous (left) and heterozygous individuals (right). White arrows indicate the amplicon from the reference *SERPINA*-BIE allele, according to GRCh38 reference genome (693 bp, 11 repetitions of 56 bp). Kb: kilobases; NTC: nontemplate control. (c) The structure of the different *SERPINA*-BIE alleles is shown. Sequence types of 56 bp are represented with boxes, and CpG sites are indicated by arrows. The cg08257009 is indicated by green arrows. Sequence types that contain two CpGs are coloured in red, while those that contain three or four CpGs are in blue. For specific analysis, alleles were grouped as low or high CpG number (CpG_N_), as indicated.

The genomic region spanning the *SERPINA*-BIE element (Figure 1(a)) was amplified by PCR, using primers (Table 1) and conditions described in the Methods section. Overall, 905 out of 917 individuals (98.7%) were successfully genotyped at the first attempt. The PCR-based genotyping assay allowed the characterization of nine different *SERPINA*-BIE alleles, according to the length of their respective PCR products. Each allele was named considering the number of 56-bp repetitions, taking as standard the size of the amplicon obtained from the reference allele (693-bp and 11 repetitions) (Figure 1(b)).

*SERPINA*-BIE alleles were sequenced to characterize their specific 56-bp repetition pattern and to determine their number of CpG sites (CpG_N_). Thirteen different 56-bp repetition types were found according to their particular sequences (Table S1). Sequence types 1–6 contain two CpGs, while four CpGs were detected in sequence types 7–12, and three in sequence type 13. Moreover, each *SERPINA*-BIE allele showed a specific combination of sequence types (Figure 1(c) and Table S2). Alleles 8–12 showed three or less sequence types with four CpGs each, thus containing 20, 22, 26, 28, and 30 CpG sites, respectively. On the other hand, alleles 13–16 showed at least five sequence types with four CpGs each, and their number of CpGs was higher (38, 42, 40, and 43 CpG sites, respectively). Considering the total CpG amount, alleles 8–12 were joined in a group of alleles with low CpG_N_, while alleles 13–16 were combined in the group of high CpG_N_ (Figure 1(c)). Interestingly, the cg08257009, previously associated with changes in lung function [26], was mapped at the third sequence type (second CpG site), which was present in all *SERPINA*-BIE alleles (Table S1 and Figure 1(c)).

### 3.2. *SERPINA*-BIE Alleles Are Asymmetrically Distributed Among Asthmatic Patients With Different *SERPINA1* Genotypes

Demographic and clinical characteristics were calculated for asthmatic patients and newborns, and, according to previous results [23], differences were detected only for women representation, *SERPINA1 Pi*∗*MM* genotype distribution, and *Pi*∗*Z* allele frequency (Table S3). HWE tests confirm that the *SERPINA*-BIE locus was in HWE, both for asthmatic patients (*p* value = 0.917) and newborns (*p* value = 0.848). Interestingly, a specific distribution of *SERPINA*-BIE allele frequencies was observed inside each group (Table 2), since *SERPINA*-BIE allele 10 was 1.3-fold increased among asthmatic patients than in newborns. Allele 15 seemed to be less frequent among asthmatic patients, but this difference was not supported after multiple comparison corrections. When the CpG content of the *SERPINA*-BIE allele was compared, alleles with high CpG_N_ were significantly more frequent among newborns (Table 2).

Descriptive statistics were also calculated independently for individuals with *Pi*∗*MM*, *Pi*∗*MS*, or *Pi*∗*MZ SERPINA1* genotypes. As expected, *Pi*∗*MM* asthmatic patients showed higher AAT levels than *Pi*∗*MZ* and *Pi*∗*MS* individuals, while no differences were found for any other variable (Table S4). HWE tests were carried out for the *SERPINA*-BIE locus, stratified by *SERPINA1* genotypes. Considering a *Bonferroni* correction (*p* value = 0.0056), HWE was confirmed for *Pi*∗*MM* (*p* value = 0.933), *Pi*∗*MZ* (*p* value = 0.067), and *Pi*∗*MS* (*p* value = 0.025) asthmatic patients. Among newborns, while the *SERPINA*-BIE locus was in HWE for *Pi*∗*MM* (*p* value = 0.856) and *Pi*∗*MZ* (*p* value = 0.015) individuals, HWE departure was detected for *PI*∗*MS* newborns (*p* value = 5.16 × 10^−4^).

Asthmatic patients with *Pi*∗*MM* genotype showed higher frequencies of *SERPINA*-BIE alleles 11 and 16, compared with individuals with *Pi*∗*MS* genotypes, while *Pi*∗*MZ* carriers exhibited higher frequencies of alleles 12 and 13. Interestingly, allele 14 was much more abundant between *Pi*∗*MS* individuals (Table 3). Among newborns, we observed a similar distribution of allele frequencies, since *SERPINA*-BIE allele 11 was more frequent in *Pi*∗*MM* individuals, alleles 12 and 13 were more abundant in newborns with *Pi*∗*MZ* genotype, and allele 14 was enriched in *Pi*∗*MS* individuals (Table 3). When patients with *Pi*∗*MS* genotypes were compared with *Pi*∗*MZ* carriers, alleles 12 (*p* value = 1.30 × 10^−03^) and 13 (*p* value = 2.52 × 10^−08^) were more frequent in *Pi*∗*MZ,* while allele 14 was more frequent between *PI*∗*MS* (*p* value = 2.35 × 10^−05^). Indeed, *SERPINA*-BIE allele 14 was present in more than 50% of all *Pi*∗*MS* individuals, both for asthmatic patients and newborns. Overall, these findings support a potential association between specific *SERPINA*-BIE alleles and *SERPINA1* genotypes, especially between *SERPINA*-BIE allele 14 and *SERPINA1 Pi*∗*MS* genotype, but also between *SERPINA*-BIE alleles 12–13 and *Pi*∗*MZ.*

**Figure 2:**
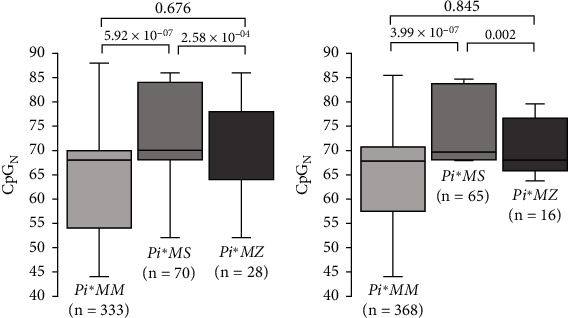
Comparison of the number of CpG sites found at *SERPINA*-BIE locus between individuals with different *SERPINA1* genotypes. (a) Sum of CpG number from both alleles (median and interquartile range) of *SERPINA*-BIE locus in asthmatic patients. (b) Sum of CpG sites from both alleles (median and interquartile range) of *SERPINA*-BIE locus in newborns. *SERPINA1* genotypes and number of individuals included in each group are shown below each box, and *p* values are shown above, according to the ANOVA test. *p* values of statistically significant different groups are in bold (*p* value < 0.05).

According to the *SERPINA*-BIE CpG content, *Pi*∗*MS* asthmatic patients showed higher frequencies of *SERPINA*-BIE alleles with high CpG_N_ than *Pi*∗*MM* individuals. Among newborns, the frequency of alleles with high CpG_N_ was also higher between *Pi*∗*MS* than in *Pi*∗*MM* individuals (Table 3). An ANOVA test was performed to compare the exact number of CpG sites present at the *SERPINA*-BIE locus. For both asthmatic patients and newborns, the CpG_N_ at the *SERPINA*-BIE locus was higher in *Pi*∗*MS* individuals when compared with *Pi*∗*MM* or *Pi*∗*MZ* patients (Figures 2(a) and 2(b)). Therefore, these results support that *Pi*∗*MS* individuals have more *SERPINA*-BIE alleles with high CpG_N_ than those with the *Pi*∗*MM* genotype.

### 3.3. Associations of *SERPINA*-BIE Alleles and CpG Content With AAT Levels Are Cofounded by *SERPINA1* Genotypes

**Figure 3:**
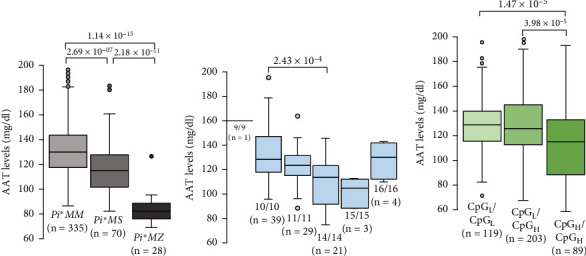
Comparison of AAT levels between asthmatic individuals with different genotypes at *SERPINA1* gene or *SERPINA*-BIE locus. (a) AAT levels (median and interquartile range) of asthmatic patients with the indicated *SERPINA1* genotypes. (b) AAT levels (median and interquartile range) of patients with the indicated *SERPINA*-BIE homozygous genotypes. (c) AAT levels (median and interquartile range) for asthmatic patients with different dosage of high CpG_N_*SERPINA*-BIE alleles. Genotypes and number of individuals in each group are indicated below each box, and significant *p* values are shown above, according to the ANOVA test (*p* value < 0.05).

AAT levels were consistent with previous studies [13], being in the range of 96.5–183.1 mg/dl for *Pi*∗*MM*, 82.2–160.7 mg/dl for *Pi*∗*MS*, and 69.1–95.4 mg/dl for *Pi*∗*MZ* patients (Figure 3(a)). AAT levels were significatively lower in asthmatic patients that were homozygous for *SERPINA*-BIE allele 14, when compared with homozygous 10/10 individuals (Figure 3(b)). However, no significant differences of AAT levels were detected when the other groups of *SERPINA*-BIE homozygous individuals were tested (Figure 3(b)). On the other hand, AAT levels were compared between patients with different dosage of *SERPINA*-BIE high CpG_N_ alleles, and results showed that AAT levels were significatively lower in individuals that carry two copies of high CpG_N_ alleles (Figure 3(c)). Overall, these results suggest that additional copies of allele 14, or other alleles with high CpG_N_, are associated with lower AAT levels. However, 16 out of 21 homozygous individuals detected for *SERPINA*-BIE allele 14 were also *Pi*∗*MS*, and the remaining five were *Pi*∗*SS*. Therefore, the observed association of *SERPINA*-BIE-specific alleles and their CpG content with and AAT levels could actually reflect the reduction of AAT levels caused by the *SERPINA1 Pi*∗*S* allele.

To test this hypothesis, the association between the number of each *SERPINA*-BIE allele with AAT levels was tested, using allele-additive linear regression models. Models were initially adjusted by age, sex, and PCs of genetic ancestry and then conditioned considering *SERPINA1* genotypes (Table 4). *SERPINA*-BIE alleles 11–14 initially exhibited statistically significant associations with AAT levels in models corrected by age and sex. However, when *SERPINA1* genotypes were included as covariates, the associations did not remain significant. Similar results were observed for CpG_N_ at the *SERPINA*-BIE locus, as well as when allele-additive models for *SERPINA*-BIE alleles with high CpG_N_ were tested (Table 4). Overall, these results support a cofounding role of *SERPINA1* genotypes in the association between AAT levels and *SERPINA*-BIE alleles. However, the limited sample number for *Pi*∗*MS* (*n* = 70) and *Pi*∗*MZ* (*n* = 28) individuals, compared with *Pi*∗*MM* (*n* = 335), could be also the cause underlying this loss of association.

### 3.4. *SERPINA1* Genotypes Are Associated With Specific *SERPINA*-BIE Alleles

In order to explore the possible association of *SERPINA*-BIE alleles with specific *SERPINA1* genotypes, asthmatic patients were grouped in *Pi*∗*MM*, *Pi*∗*MS*, and *Pi*∗*MZ*, while individuals with other *SERPINA1* genotypes were excluded due to their small sample size (Table S4). When the copy number of each *SERPINA*-BIE allele was compared between *Pi*∗*MM* and *Pi*∗*MS* patients, results showed differences for alleles 10, 11, 14, 15, and 16 (Table 5). *SERPINA*-BIE alleles 10, 11, 15, and 16 showed OR < 1, which means that each additional copy of these alleles reduces the probability of being a carrier of *Pi*∗*S* allele, between 1.8 and 3.8 odds. On the other hand, each additional copy of the *SERPINA*-BIE allele 14 was associated with the *Pi*∗*MS* genotype with an OR of 14.8. Therefore, each additional copy of *SERPINA*-BIE allele 14 drastically increases the probability of carrying the *Pi*∗*S* allele. Except for *SERPINA*-BIE allele 16, results were similar for newborns, but in this group, each additional copy of allele 14 was associated with 7.6 times more odds of being a *Pi*∗S carrier.

Moreover, a strong association was found between the CpG_N_ at the *SERPINA*-BIE locus and the *Pi*∗*MS* genotype, showing that each additional CpG site at the *SERPINA*-BIE locus increases 1.08 odds the probability to be a *Pi*∗*S* carrier, both among patients and newborns (Table 5). When *SERPINA*-BIE alleles were combined according to their CpG_N_, each additional *SERPINA*-BIE allele with a high CpG_N_ (alleles 13–16) increases 3.7 odds the probability to be a *Pi*∗*S* allele carrier among asthmatic patients, and 2.9 folds among newborns.

When patients with the *Pi*∗*MM* genotype were compared with the *Pi*∗*MZ* group, results showed differences for alleles 11 and 13 (Table 5). While each additional copy of *SERPINA*-BIE allele 11 decreases 3.7 odds the probability to be a carrier of *SERPINA1 Pi*∗*Z*, each additional copy of the *SERPINA*-BIE allele 13 was positively associated with being *Pi*∗*MZ*. However, this OR value is overestimated and actually reflects the low frequency of allele 13 among *Pi*∗*MM* individuals or could be inflated due to the low number of *Pi*∗*MZ* individuals (Table 3). However, as allele 13 (and also allele 12) also showed a statistically significant association with the *Pi*∗*MZ* genotype in newborns, with a positive OR value, this result suggests that this allele is strongly associated with the *Pi*∗*MZ* genotype. Finally, models that combine alleles according to their CpG_N_ were statistically significative for asthmatic patients but were not replicated when the exact number of CpGs was considered, neither in newborns (Table 5).

Overall, these results support an association between *SERPINA1*-BIE allele 14 and *SERPINA1 Pi*∗*S* allele and also suggest an association between allele 13 and *Pi*∗*Z*. These associations were observed among asthmatic patients and replicated in the general population. Moreover, the CpG content of the *SERPINA*-BIE locus has been strongly associated with the *SERPINA1 Pi*∗*MS* genotype, which means that higher CpG content at this locus increases the probability to be a *Pi*∗*S* allele carrier. However, in the case of the *SERPINA1 Pi*∗*Z* allele, this result was partially not replicated, but it is probably the reflection of the reduced sample number for the *Pi*∗*MZ* group.

## 4. Discussion

The PCR-based genotyping assay developed in the present study, combined with the fast alkaline-extraction method [31], allowed the molecular characterization of the *SERPINA*-BIE locus from 917 individuals, including asthmatic patients and newborns. Results showed that the structure of the *SERPINA*-BIE locus is structurally complex, including at least 13 different sequence types of 56 bp, which were combined to conform a minimum of nine different structural variants (alleles). Moreover, different *SERPINA*-BIE alleles showed specific CpG patterns, with specific CpG content. Since alleles with a higher number of 56-bp repetitions are richer in CpG sites, we suggest that this region could function as a regulatory element over the enhancer activity.

It has been observed that the number of CpG sites present in different enhancers can regulate gene expression in various ways. Recent studies have shown that most CpG islands distant to promoters (orphan CpG islands) display chromatin features that resemble to active enhancers and that enhancers associated to these CpG islands usually show stronger activity, are broadly expressed, and are highly conserved [40, 41]. In addition, the CpG density of enhancers seems to play a major role in determining their regulatory activity [42], and CpG-based epigenetic regulation has been proposed as a key element for the enhancer recognition by activator proteins [43] and is able to control long-range chromatin interactions [44]. Another study found that most eQTM (expression Quantitative Trait Methylation) loci in childhood asthma were located in enhancer regions, affecting gene expression in lung tissue [45]. Therefore, *SERPINA*-BIE could play an important role in the regulation of the *SERPINA* gene cluster expression, including the *SERPINA1* gene, and its activity could be affected by the specific CpG content found in the different alleles. Unfortunately, DNA preparations used in the present work were not suitable for the detection of the methylation profile of *SERPINA*-BIE, and this hypothesis should be tested in the future.

Overall, our results strongly support an association between specific *SERPINA*-BIE alleles and/or their CpG content, with certain *SERPINA1* genotypes. Therefore, the *SERPINA*-BIE locus could be explored in the future as a possible biomarker of COPD and emphysema prognosis for *Pi*∗*S* and *Pi*∗*Z* carriers since these alleles have been classically associated with these diseases [46]. Moreover, since *Pi*∗*S* and *Pi*∗*Z* alleles have been recently associated with asthma exacerbations [23], genotyping of the *SERPINA1-*BIE locus could also be investigated as a risk stratification tool for asthma exacerbations in the future.

Moreover, we have tested for the first time the association between AAT levels with *SERPINA*-BIE alleles and with their CpG content. AAT levels were significantly associated with the copy number of *SERPINA*-BIE alleles 11, 12, 13, and 14. However, when models were adjusted considering *SERPINA1* genotypes, the associations did not remain significant. These results represent an excellent example of how genome-wide associations could be misinterpreted, since they can be the synthetic result of other genomic regions with real functional implications [47]. In this context, the association proposed for the differential methylation of cg08257009 (one of the CpGs placed at *SERPINA*-BIE), with the FEV_1_/FVC ratio in adult smokers, remains significant after correction with the *SERPINA1* genotype [26]. Therefore, the methylation status of *SERPINA1*-BIE should be studied at the sequence level, considering the complex structure of this locus revealed in the present work.

We found a strong association between *SERPINA*-BIE allele 14 and *SERPINA1 Pi*∗*MS* genotype, and to a lesser extent for allele 13 with *Pi*∗*MZ*, among asthmatic patients. However, it would be interesting to recruit more *Pi*∗*MS* and *Pi*∗*MZ* individuals, to homogenize the sample size regarding the *Pi*∗*MM* group. These findings were assessed for replication in the general population of La Palma island (newborns), and both associations were confirmed. Our study proposes that *SERPINA1 Pi*∗*MS* asthmatic patients have 14.8 more probability to be carriers of *SERPINA*-BIE allele 14 than *Pi*∗*MM* individuals. Indeed, the *Pi*∗*S* allele was almost exclusively found combined with *SERPINA*-BIE allele 14. Overall, these results suggest that the *SERPINA*-BIE regulatory activity over the *SERPINA1* gene could be different when *Pi*∗*M*, *Pi*∗*S*, or *Pi*∗*Z* alleles are present, since its CpG content changes accordingly. However, it is necessary to perform functional experiments in the future to evaluate this hypothesis. These findings are relevant, since they provide new perspectives about distal regulation of *SERPINA1* gene expression and could contribute to the understanding of the molecular mechanisms involved in AATD-associated diseases.

In conclusion, this study has provided insights into the understanding of molecular mechanisms involved in AATD characterizing, at the sequence level, an additional genomic distal regulator that could influence the expression of the *SERPINA1* gene. Although it is still early to apply these findings in routine clinical practice, the methods developed in this work could facilitate AATD prognosis in the future.

## 5. Conclusions

After characterization of the *SERPINA*-BIE locus (LOC126862032), 13 different types of 56-bp motif were described, which are combined in at least nine different structural variants (alleles) of this locus. Interestingly, each allele showed a specific CpG content, and specific alleles were associated with *SERPINA1 Pi*∗*Z* (rs28929474) and *Pi*∗*S* (rs17580) variants.

## Figures and Tables

**Table 1:** Primer sequences used for *SERPINA*-BIE genotyping and Sanger's sequencing.

**Primer ID**	**Sequence (5**′**-3**′**)**	**Genomic region** ^a^	**Tm** ^b^	**Use**
AATE-F5	TCTTCCAGCTCAGGGTTTCTCAG	Chr14:94346596-94346618	65.2°C	PCR
AATE-R5	TGCTGCTGGCATCCAATAGG	Chr14:94345926-94345945	63.6°C	
AATE-SF5	CTCAGGGTTTCTCAGCCTCATC	Chr14:94346589-94346610	64.0°C	Sequencing

^a^According to GRCh38 reference genome version.

^b^Melting temperature (Tm) was predicted with GeneRunner software.

**Table 2:** *SERPINA*-BIE allele frequencies in asthmatic patients and newborns.

***SERPINA*-BIE allele**	**Asthmatic patients (** **n** = 450**)**^a^	**Newborns (** **n** = 455**)**	**p** ** value** ^b^
8	0 (0.000)	4 (0.004)	0.125
9	7 (0.008)	7 (0.008)	1.000
10	248 (0.276)	198 (0.218)	**0.005**
11	223 (0.248)	204 (0.224)	0.260
12	4 (0.004)	5 (0.005)	1.000
13	33 (0.037)	24 (0.026)	0.263
14	190 (0.211)	223 (0.245)	0.096
15	96 (0.107)	131 (0.144)	0.020
16	99 (0.110)	114 (0.125)	0.350
High CpG_N_^c^	418 (0.464)	492 (0.541)	**0.001**

*Note:* Bold *p* values for allele 10 (0.005) and high CpGN (0.001) rows are significant.

^a^Counts and frequencies for each *SERPINA*-BIE allele (between brackets), obtained for the indicated number of genotyped individuals (*n*).

^b^Differences between groups were evaluated with the chi-squared test, including a Bonferroni correction, *p* value = 0.0056 (0.05/9 allele comparisons).

^c^ *SERPINA*-BIE alleles with high CpG_N_ (alleles 13–16).

**Table 3:** Comparison of *SERPINA*-BIE allele frequencies among individuals with *SERPINA1 Pi*∗*MM*, *Pi*∗*MS*, and *Pi*∗*MZ* genotypes.

***SERPINA*-BIE alleles**	**Asthmatic patients**					**Newborns**				
	**MM (** **n** = 333**)**^a^	**MS (** **n** = 70**)**	**p** ** value** ^b^	**MZ (** **n** = 28**)**	**p** ** value**	**MM (** **n** = 368**)**	**MS (** **n** = 65**)**	**p** ** value**	**MZ (** **n** = 16**)**	**p** ** value**
8	0.000	0.000	1.000	0.000	1.000	0.005	0.000	0.888	0.000	1.000
9	0.011	0.000	0.612	0.000	1.000	0.010	0.000	0.603	0.000	1.000
10	0.306	0.193	0.009	0.268	0.653	0.238	0.154	0.046	0.094	0.094
11	0.294	0.150	**6.9 ×10^−04^**	0.107	0.004	0.250	0.115	**0.001**	0.156	0.319
12	0.000	0.000	1.000	0.054	**4.4 × 10^−04^**	0.001	0.000	1.000	0.125	**1.2 × 10^−05^**
13	0.002	0.014	0.080	0.375	**2.2 × 10^−16^**	0.011	0.008	1.000	0.375	**2.2 × 10^−16^**
14	0.129	0.536	**2.2 × 10^−16^**	0.089	0.514	0.189	0.562	**2.2 × 10^−16^**	0.063	0.099
15	0.123	0.064	0.064	0.054	0.135	0.159	0.077	0.021	0.125	0.805
16	0.135	0.043	**0.003**	0.054	0.096	0.137	0.085	0.132	0.063	0.296
High CpG_N_^c^	0.389	0.657	**1.0 × 10^−08^**	0.571	0.011	0.496	0.731	**1.2 × 10^−06^**	0.625	0.212

*Note:* All *p* values that are significant are presented in bold.

^a^Frequencies for each *SERPINA*-BIE allele, obtained for the indicated number of *Pi*∗*MM*-, *Pi*∗*MS*-, or *Pi*∗*MZ*-genotyped individuals (*n*).

^b^Differences between *Pi*∗*MM* and *Pi*∗*MS* or among *Pi*∗*MM* and *Pi*∗*MZ* were evaluated with the chi-squared test, including a Bonferroni correction, *p* value = 0.0056 (0.05/9 allele comparisons).

^c^ *SERPINA*-BIE alleles with high CpG_N_ (alleles 13–16).

**Table 4:** Results of linear regression models to test the association between AAT levels and *SERPINA*-BIE alleles or CpG number.

***SERPINA*-BIE allele**	**Not adjusted**	**A** **g** **e** + **sex** + **PCs**	**A** **g** **e** + **sex** + **PCs** + **SERPINA**1**genotype**^c^
9	0.549 (5.442 ± 9.075)	0.675 (3.710 ± 8.839)	0.809 (−1.626 ± 6.727)
10	**0.009** (5.230 ± 2.006)	0.067 (4.010 ± 2.180)	0.915 (0.180 ± 1.689)
11	**0.002** (6.460 ± 2.112)	**0.004** (6.514 ± 2.246)	0.853 (−0.333 ± 1.793)
12	3.49 × 10^−04^ (−48.267 ± 13.383)	**0.004** (−54.030 ± 18.471)	0.343 (−14.229 ± 14.979)
13	<2.00 × 10^−16^ (−49.326 ± 4.425)	<2.00 × 10^−16^ (−48.607 ± 4.846)	0.442 (6.212 ± 8.068)
14	6.66 × 10^−04^ (−7.681 ± 2.240)	**0.004** (−7.002 ± 2.442)	0.173 (3.191 ± 2.338)
15	0.576 (1.738 ± 3.109)	0.320 (3.353 ± 3.367)	0.190 (−3.410 ± 2.596)
16	**0.023** (6.979 ± 3.050)	0.151 (4.636 ± 3.226)	0.935 (−0.203 ± 2.490)
CpG_N_^a^	1.87 × 10^−04^ (−0.464 ± 0.123)	**0.001** (−0.424 ± 0.132)	0.787 (0.030 ± 0.109)
High CpG_N_^b^	1.70 × 10^−06^ (−8.926 ± 1.837)	4.02 × 10^−05^ (−8.283 ± 1.989)	0.813 (0.400 ± 1.693)

*Note:* For each linear regression analysis, statistically significant *p* values are depicted in boldface (*p* value < 0.05), while beta‐values ± standard deviations are shown between brackets.

^a^Models include the specific number of CpG sites detected for each individual at *SERPINA*-BIE locus (sum of CpG_N_ from both *SERPINA*-BIE alleles).

^b^Allele-additive model, considering the copy number of high CpG_N_ alleles (alleles 13–16).

^c^Models were additionally adjusted with *SERPINA1* genotypes (*Pi*∗*MM*, *Pi*∗*MS*, and *Pi*∗*MZ*).

**Table 5:** Results of logistic regression models to test the associations of *SERPINA1* genotypes with *SERPINA*-BIE alleles and their CpG content.

***SERPINA*-BIE allele** ^a^	***Pi*** ∗***MM* vs. *Pi***∗***MS***^d^		***Pi*** ∗***MM* vs. *Pi***∗***MZ***^d^	
	**Asthmatic patients**	**Newborns**	**Asthmatic patients**	**Newborns**
10	**0.037** (0.56 [0.32–0.96])	**0.038** (0.58 [0.35–0.97])	0.466 (0.77 [0.39–1.54])	0.075 (0.34 [0.10–1.12])
11	**0.003** (0.40 [0.22–0.73])	7.07 × 10^−04^ (0.37 [0.21–0.66])	**0.008** (0.27 [0.10–0.71])	0.216 (0.53 [0.20–1.44])
12	NA	NA	NA	3.45 × 10^−05^ (121.07 [12.51–1171.98])
13	0.060 (11.65 [0.91–149.04])	0.773 (0.73 [0.09–6.00])	3.19 × 10^−08^ (1534.53 [113.99–20658.47])	6.36 × 10^−13^ (137.46 [35.93–525.80])
14	6.77 × 10^−13^ (14.81 [7.10–30.91])	3.47 × 10^−14^ (7.58 [4.49–12.79])	0.824 (0.89 [0.34–2.38])	0.100 (0.30 [0.07–1.26])
15	**0.041** (0.38 [0.15–0.96])	**0.018** (0.44 [0.23–0.87])	0.075 (0.16 [0.02–1.21])	0.615 (0.76 [0.26–2.20])
16	**0.015** (0.26 [0.09–0.77])	0.116 (0.59 [0.30–1.14])	0.270 (0.50 [0.14–1.72])	0.238 (0.42 [0.10–1.78])
CpG_N_^b^	2.87 × 10^−06^ (1.08 [1.05–1.12])	3.76 × 10^−07^ (1.08 [1.05–1.11])	0.090 (1.04 [0.99–1.08])	0.447 (1.02 [0.97–1.07])
High CpG_N_^c^	9.16 × 10^−07^ (3.68 [2.19–6.18])	1.10 × 10^−06^ (2.96 [1.91–4.58])	**0.004** (2.78 [1.40–5.54])	0.144 (1.74 [0.83–3.68])

*Note:* Statistically significant *p* values are depicted in boldface (*p* value < 0.05) for each logistic regression model, while the odds ratio (OR) and its corresponding 95% confidence interval are shown between brackets.

^a^Allele-additive models, considering the number of each *SERPINA*-BIE allele (only alleles 10–16 were considered, since the number of individuals with alleles 8–9 were limited).

^b^Models include the specific CpG_N_ detected in each individual for both *SERPINA*-BIE alleles.

^c^Allele-additive model, considering alleles with high CpG_N_ (alleles 13–16).

^d^Age, biological sex, and principal components of genetic ancestry were used as covariates in the logistic regression models for asthmatic patients, or only biological sex for newborns.

## Data Availability

The datasets analyzed during the current study are available under reasonable request to the corresponding author.

## Supporting Information

Supporting Information Additional supporting information can be found online in the Supporting Information section. Table S1. Sequences obtained for the different 56-bp sequence types found at the *SERPINA*-BIE locus. Table S2. Sequences of the different *SERPINA*-BIE alleles. Table S3. Clinical and demographic characteristics of the individuals recruited in this study. Table S4. Clinical and demographic characteristics of analyzed individuals, considering *SERPINA1* genotypes.
