# Supplementary material for: Pi∗S and Pi∗Z Alleles of SERPINA1 Gene Are Associated With Specific Variants of a BRD4-Independent Enhancer
Source: Hum Mutat. 2024 Jun 25;2024:6472805. doi: 10.1155/2024/6472805 (PMC11924960; doi:10.1155/2024/6472805)
Supplement: Supporting Information — Additional supporting information can be found online in the Supporting Information section. Table S1. Sequences obtained for the different 56-bp sequence types found at the SERPINA-BIE locus. Table S2. Sequences of the different SERPINA-BIE alleles. Table S3. Clinical and demographic characteristics of the individuals recruited in this study. Table S4. Clinical and demographic characteristics of analyzed individuals, considering SERPINA1 genotypes. [file 6472805.f1.pdf]

*Pi*S* and *Pi*Z* alleles of *SERPINA1* gene are associated with specific variants of a BRD4-Independent Enhancer

Ainhoa Escuela-Escobar, Esther Herrera-Luis, Elena Martín-González, José María Hernández-Pérez, Mario A. González Carracedo, and José Antonio Pérez Pérez

Table Supplementary 1. Sequences obtained for the different 56-bp sequence-types found at *SERPINA* -BIE locus.

Sequence-types	Sequences	Number of CpG
1	CACATCA**CG**GGCTGGAAAGTCCTGTGTGGTTCAGGGGCTTTCC**CG**TGCACTGTAGG	2
2	CACATCA**CG**GGCTGGAAAGTCCTGTGTGGTTCAGGGGCTTTCC**CG**TGCACTGCAGG	2
3	CACATCA**CG**GGCTGGAAATTCCTGTGTGGTGCAGGGCCTTTCC**CG**TGCACTGCAGG	2
4	CACATCA**CG**GGCTGGACAGTCCTGTGTGGTGCAGGGGCTTTCC**CG**TGCACTGCAGG	2
5	CACATCA**CG**GGCTGGAAAGTCCTGTGTGGTTCAGGGGCTTTC**CG**GTGCACTGCAGG	2
6	CACATCA**CG**GGCTGGAAAGTCCTGTGTGGTGCAGGGGCTTTCC**CG**TGCACTGCAGG	2
7	CACATCA**CG**GGCTGGA**CG**GTCCTG**CG**TGGTGCAGGGGCTTTCC**CG**TGCACTGCAGG	4
8	CACATCA**CG**GGCTGGA**CG**GTCCTG**CG**TGGTGCAGGGGCTTTCC**CG**TGCATTGTAGG	4
9	CACATCA**CG**GGCTGGA**CG**GTCCTG**CG**TGGTGCAGGGGCTTTCC**CG**TGCATTGTAAG	4
10	CACATCA**CG**GGCTGGA**CG**GTCCTG**CG**TGGTTCAGGGGCTTTCC**CG**TGCACTGCAGG	4
11	CACATCA**CG**GGCTGGA**CG**GTCCTG**CG**TGGTTCAGGGGCTTTCC**CG**TGCACTGCAAG	4
12	CACATCA**CG**GGCTGGA**CG**GTCCTG**CG**TGGTGCAGGGGCTTTCC**CG**TGCACTGCAAG	4
13	CACATCA**CG**GGCTGGA**CG**GTCCTGCCTGGTGCAGGGGCTTTCC**CG**TGCACTGCAAG	3

*Pi**S* and *Pi**Z* alleles of *SERPINA1* gene are associated with specific variants of a BRD4-Independent Enhancer

Ainhoa Escuela-Escobar, Esther Herrera-Luis, Elena Martín-González, José María Hernández-Pérez, Mario A. González Carracedo, and José Antonio Pérez Pérez

Table Supplementary 2. Sequences of the different *SERPINA*-BIE alleles.

***SERPINA*-BIE alleles**	**Sequence**
**8**	CACATCACGGGCTGGAAAGTCCTGTGTGGTTCAGGGGCTTTCCCGTGCACTGTAGGCACATCACGGGCTGGAAAGTCCTGTGTGGTTCAGG<br>GGCTTTCCCGTGCACTGCAGGCACATCACGGGCTGGAAATTCCTGTGTGGTGCAGGGCCTTTCCCGTGCACTGCAGGCACATCACGGGCT<br>GGAAAGTCCTGTGTGGTTCAGGGGCTTTCCGGTGCACTGCAGGCACATCACGGGCTGGAAAGTCCTGTGTGGTGCAGGGGCTTTCCCGTGC<br>ACTGCAGGCACATCACGGGCTGGAAAGTCCTGTGTGGTGCAGGGGCTTTCCCGTGCACTGCAGGCACATCACGGGCTGGACGGTCCTGCG<br>TGGTTCAGGGGCTTTCCCGTGCACTGCAGGCACATCACGGGCTGGACGGTCCTGCGTGGTGCAGGGGCTTTCCCGTGCATTGTAGG
**9**	CACATCACGGGCTGGAAAGTCCTGTGTGGTTCAGGGGCTTTCCCGTGCACTGTAGGCACATCACGGGCTGGAAAGTCCTGTGTGGTTCAGG<br>GGCTTTCCCGTGCACTGCAGGCACATCACGGGCTGGAAATTCCTGTGTGGTGCAGGGCCTTTCCCGTGCACTGCAGGCACATCACGGGCT<br>GGACAGTCCTGTGTGGTGCAGGGGCTTTCCCGTGCACTGCAGGCACATCACGGGCTGGACAGTCCTGTGTGGTGCAGGGGCTTTCCCGTG<br>CACTGCAGGCACATCACGGGCTGGAAAGTCCTGTGTGGTTCAGGGGCTTTCCGGTGCACTGCAGGCACATCACGGGCTGGAAAGTCCTGTG<br>TGGTGCAGGGGCTTTCCCGTGCACTGCAGGCACATCACGGGCTGGACGGTCCTGCGTGGTGCAGGGGCTTTCCCGTGCACTGCAGGCACA<br>TCACGGGCTGGACGGTCCTGCGTGGTGCAGGGGCTTTCCCGTGCATTGTAGG
**10**	CACATCACGGGCTGGAAAGTCCTGTGTGGTTCAGGGGCTTTCCCGTGCACTGTAGGCACATCACGGGCTGGAAAGTCCTGTGTGGTTCAGG<br>GGCTTTCCCGTGCACTGCAGGCACATCACGGGCTGGAAATTCCTGTGTGGTGCAGGGCCTTTCCCGTGCACTGCAGGCACATCACGGGCT<br>GGACAGTCCTGTGTGGTGCAGGGGCTTTCCCGTGCACTGCAGGCACATCACGGGCTGGACAGTCCTGTGTGGTGCAGGGGCTTTCCCGTG<br>CACTGCAGGCACATCACGGGCTGGAAAGTCCTGTGTGGTTCAGGGGCTTTCCGGTGCACTGCAGGCACATCACGGGCTGGAAAGTCCTGTG<br>TGGTGCAGGGGCTTTCCCGTGCACTGCAGGCACATCACGGGCTGGACGGTCCTGCGTGGTGCAGGGGCTTTCCCGTGCACTGCAGGCACA<br>TCACGGGCTGGACGGTCCTGCGTGGTGCAGGGGCTTTCCCGTGCACTGCAGGCACATCACGGGCTGGACGGTCCTGCGTGGTGCAGGGGC<br>TTTCCCGTGCATTGTAGG

SERPINA- BIE alleles	Sequence
**11**	CACATCACGGGCTGGAAAGTCCTGTGTGGTTCAGGGGCTTTCCCGTGCACTGTAGGCACATCACGGGCTGGAAAGTCCTGTGTGGTTCAGG<br>GGCTTTCCCGTGCACTGCAGGCACATCACGGGCTGGAAATTCCTGTGTGGTGCAGGGCCTTTCCCGTGCACTGCAGGCACATCACGGGCT<br>GGACAGTCCTGTGTGGTGCAGGGGCTTTCCCGTGCACTGCAGGCACATCACGGGCTGGACAGTCCTGTGTGGTGCAGGGGCTTTCCCGTG<br>CACTGCAGGCACATCACGGGCTGGACAGTCCTGTGTGGTGCAGGGGCTTTCCCGTGCACTGCAGGCACATCACGGGCTGGAAAGTCCTGT<br>GTGGTTCAGGGGCTTTCCGGTGCACTGCAGGCACATCACGGGCTGGAAAGTCCTGTGTGGTGCAGGGGCTTTCCCGTGCACTGCAGGCAC<br>ATCACGGGCTGGACGGTCCTGCGTGGTGCAGGGGCTTTCCCGTGCACTGCAGGCACATCACGGGCTGGACGGTCCTGCGTGGTGCAGGGG<br>CTTTCCCGTGCACTGCAGGCACATCACGGGCTGGACGGTCCTGCGTGGTGCAGGGGCTTTCCCGTGCATTGTAAG
**12**	CACATCACGGGCTGGAAAGTCCTGTGTGGTTCAGGGGCTTTCCCGTGCACTGTAGGCACATCACGGGCTGGAAAGTCCTGTGTGGTTCAGG<br>GGCTTTCCCGTGCACTGCAGGCACATCACGGGCTGGAAATTCCTGTGTGGTGCAGGGCCTTTCCCGTGCACTGCAGGCACATCACGGGCT<br>GGACAGTCCTGTGTGGTGCAGGGGCTTTCCCGTGCACTGCAGGCACATCACGGGCTGGACAGTCCTGTGTGGTGCAGGGGCTTTCCCGTG<br>CACTGCAGGCACATCACGGGCTGGACAGTCCTGTGTGGTGCAGGGGCTTTCCCGTGCACTGCAGGCACATCACGGGCTGGACAGTCCTGT<br>GTGGTGCAGGGGCTTTCCCGTGCACTGCAGGCACATCACGGGCTGGAAAGTCCTGTGTGGTTCAGGGGCTTTCCGGTGCACTGCAGGCAC<br>ATCACGGGCTGGAAAGTCCTGTGTGGTGCAGGGGCTTTCCCGTGCACTGCAGGCACATCACGGGCTGGACGGTCCTGCGTGGTGCAGGGG<br>CTTTCCCGTGCACTGCAGGCACATCACGGGCTGGACGGTCCTGCGTGGTGCAGGGGCTTTCCCGTGCACTGCAGGCACATCACGGGCTGG<br>ACGGTCCTGCGTGGTGCAGGGGCTTTCCCGTGCATTGTAGG
**13**	CACATCACGGGCTGGAAAGTCCTGTGTGGTTCAGGGGCTTTCCCGTGCACTGTAGGCACATCACGGGCTGGAAAGTCCTGTGTGGTTCAGG<br>GGCTTTCCCGTGCACTGCAGGCACATCACGGGCTGGAAATTCCTGTGTGGTGCAGGGCCTTTCCCGTGCACTGCAGGCACATCACGGGCT<br>GGACAGTCCTGTGTGGTGCAGGGGCTTTCCCGTGCACTGCAGGCACATCACGGGCTGGACAGTCCTGTGTGGTGCAGGGGCTTTCCCGTG<br>CACTGCAGGCACATCACGGGCTGGACAGTCCTGTGTGGTGCAGGGGCTTTCCCGTGCACTGCAGGCACATCACGGGCTGGACAGTCCTGT<br>GTGGTGCAGGGGCTTTCCCGTGCACTGCAGGCACATCACGGGCTGGACGGTCCTGCGTGGTGCAGGGGCTTTCCCGTGCACTGCAGGCAC<br>ATCACGGGCTGGACGGTCCTGCGTGGTGCAGGGGCTTTCCCGTGCACTGCAGGCACATCACGGGCTGGACGGTCCTGCGTGGTGCAGGGG<br>CTTTCCCGTGCACTGCAGGCACATCACGGGCTGGACGGTCCTGCGTGGTGCAGGGGCTTTCCCGTGCACTGCAGGCACATCACGGGCTGG<br>ACGGTCCTGCGTGGTGCAGGGGCTTTCCCGTGCACTGCAGGCACATCACGGGCTGGACGGTCCTGCGTGGTGCAGGGGCTTTCCCGTGCA<br>TTGTAGG

SERPINA- BIE alleles	Sequence
**14**	CACATCACGGGCTGGAAAGTCCTGTGTGGTTCAGGGGCTTTCCCGTGCACTGTAGGCACATCACGGGCTGGAAAGTCCTGTGTGGTTCAGG<br>GGCTTTCCCGTGCACTGCAGGCACATCACGGGCTGGAAATTCCTGTGTGGTGCAGGGCCTTTCCCGTGCACTGCAGGCACATCACGGGCT<br>GGACAGTCCTGTGTGGTGCAGGGGCTTTCCCGTGCACTGCAGGCACATCACGGGCTGGACAGTCCTGTGTGGTGCAGGGGCTTTCCCGTG<br>CACTGCAGGCACATCACGGGCTGGAAAGTCCTGTGTGGTTCAGGGGCTTTCCGGTGCACTGCAGGCACATCACGGGCTGGAAAGTCCTGTG<br>TGGTGCAGGGGCTTTCCCGTGCACTGCAGGCACATCACGGGCTGGACGGTCCTGCGTGGTGCAGGGGCTTTCCCGTGCACTGCAGGCACA<br>TCACGGGCTGGACGGTCCTGCGTGGTGCAGGGGCTTTCCCGTGCACTGCAGGCACATCACGGGCTGGACGGTCCTGCGTGGTGCAGGGGC<br>TTTCCCGTGCACTGCAGGCACATCACGGGCTGGACGGTCCTGCGTGGTGCAGGGGCTTTCCCGTGCACTGCAGGCACATCACGGGCTGGA<br>CGGTCCTGCGTGGTGCAGGGGCTTTCCCGTGCACTGCAGGCACATCACGGGCTGGACGGTCCTGCGTGGTGCAGGGGCTTTCCCGTGCAC<br>TGCAGGCACATCACGGGCTGGACGGTCCTGCGTGGTGCAGGGGCTTTCCCGTGCATTGTAAG
**15**	CACATCACGGGCTGGAAAGTCCTGTGTGGTTCAGGGGCTTTCCCGTGCACTGTAGGCACATCACGGGCTGGAAAGTCCTGTGTGGTTCAGG<br>GGCTTTCCCGTGCACTGCAGGCACATCACGGGCTGGAAATTCCTGTGTGGTGCAGGGCCTTTCCCGTGCACTGCAGGCACATCACGGGCT<br>GGACAGTCCTGTGTGGTGCAGGGGCTTTCCCGTGCACTGCAGGCACATCACGGGCTGGACAGTCCTGTGTGGTGCAGGGGCTTTCCCGTG<br>CACTGCAGGCACATCACGGGCTGGACAGTCCTGTGTGGTGCAGGGGCTTTCCCGTGCACTGCAGGCACATCACGGGCTGGACAGTCCTGT<br>GTGGTGCAGGGGCTTTCCCGTGCACTGCAGGCACATCACGGGCTGGACAGTCCTGTGTGGTGCAGGGGCTTTCCCGTGCACTGCAGGCAC<br>ATCACGGGCTGGACAGTCCTGTGTGGTGCAGGGGCTTTCCCGTGCACTGCAGGCACATCACGGGCTGGACAGTCCTGTGTGGTGCAGGGG<br>CTTTCCCGTGCACTGCAGGCACATCACGGGCTGGACGGTCCTGCGTGGTGCAGGGGCTTTCCCGTGCACTGCAGGCACATCACGGGCTGG<br>ACGGTCCTGCGTGGTGCAGGGGCTTTCCCGTGCACTGCAGGCACATCACGGGCTGGACGGTCCTGCGTGGTGCAGGGGCTTTCCCGTGCA<br>CTGCAGGCACATCACGGGCTGGACGGTCCTGCGTGGTGCAGGGGCTTTCCCGTGCACTGCAGGCACATCACGGGCTGGACGGTCCTGCGT<br>GGTGCAGGGGCTTTCCCGTGCATTGTAGG
**16**	CACATCACGGGCTGGAAAGTCCTGTGTGGTTCAGGGGCTTTCCCGTGCACTGTAGGCACATCACGGGCTGGAAAGTCCTGTGTGGTTCAGG<br>GGCTTTCCCGTGCACTGCAGGCACATCACGGGCTGGAAATTCCTGTGTGGTGCAGGGCCTTTCCCGTGCACTGCAGGCACATCACGGGCT<br>GGAAATTCCTGTGTGGTGCAGGGCCTTTCCCGTGCACTGCAGGCACATCACGGGCTGGAAATTCCTGTGTGGTGCAGGGCCTTTCCCGTGC<br>ACTGCAGGCACATCACGGGCTGGAAATTCCTGTGTGGTGCAGGGCCTTTCCCGTGCACTGCAGGCACATCACGGGCTGGACAGTCCTGTGT<br>GGTGCAGGGGCTTTCCCGTGCACTGCAGGCACATCACGGGCTGGACAGTCCTGTGTGGTGCAGGGGCTTTCCCGTGCACTGCAGGCACAT<br>CACGGGCTGGAAAGTCCTGTGTGGTTCAGGGGCTTTCCGGTGCACTGCAGGCACATCACGGGCTGGAAAGTCCTGTGTGGTGCAGGGGCTT<br>TCCCGTGCACTGCAGGCACATCACGGGCTGGACGGTCCTGCGTGGTTCAGGGGCTTTCCCGTGCACTGCAAGCACATCACGGGCTGGACG<br>GTCCTGCGTGGTGCAGGGGCTTTCCCGTGCACTGCAAGCACATCACGGGCTGGACGGTCCTGCGTGGTGCAGGGGCTTTCCCGTGCACTG<br>CAGGCACATCACGGGCTGGACGGTCCTGCGTGGTGCAGGGGCTTTCCCGTGCACTGCAGGCACATCACGGGCTGGACGGTCCTGCCTGGT<br>GCAGGGGCTTTCCCGTGCACTGCAAGCACATCACGGGCTGGACGGTCCTGCGTGGTGCAGGGGCTTTCCCGTGCACTGCAGG

_Pi*S_ and _Pi*Z_ alleles of _SERPINA1_ gene are associated with specific variants of a BRD4-Independent Enhancer

Ainhoa Escuela-Escobar, Esther Herrera-Luis, Elena Martín-González, José María Hernández-Pérez, Mario A. González Carracedo, and José Antonio Pérez Pérez

Table Supplementary 3. Clinical and demographic characteristics of the individuals recruited in this study.

Characteristics		Asthmatic patients (n = 452)	Newborns (n = 465)	*p*- value
**Age (years)**		47.1 (32.0 - 63.0)	NA	NA
**Sex (% female)**		292 (64.6)	217 (46.8)	**$8.12x10^{-08}$**
**BMI**		28.5 (24.0 - 32.4)	NA	NA
**FEV_1/FVC**		0.8 (0.7 - 0.9)	NA	NA
**IgE levels (UI/ml)**		282.9 (36.6 - 300.4)	NA	NA
**Eosinophil counts (cells/µl)**		296.7 (200.0 - 400.0)	NA	NA
**AAT levels (mg/dl)**		125.1 (112.4 - 138.2)	NA	NA
**Asthma control (%)**		206 (45.7)	NA	NA
***SERPINA1* genotypes**	***Pi*MM***	335 (74.1)	375 (80.6)	**0.023**
	Pi*MS	70 (15.5)	68 (14.6)	0.78
	Pi*MZ	28 (6.2)	16 (3.4)	0.07
	Pi*SS	5 (1.1)	3 (0.6)	0.50
	Pi*SZ	9 (1.9)	3 (0.6)	0.09
	Pi*ZZ	0 (0.0)	0 (0.0)	NA
	**Rare variants**	5 (1.1)	0 (0.0)	NA
***SERPINA1* alleles**	***Pi*S***	84 (18.6)	74 (15.9)	0.33
	Pi*Z	37 (8.2)	19 (4.1)	**0.01**

Continuous variables (age, BMI, FVE_1/FVC, IgE levels, eosinophil counts, and AAT levels) were summarized with the median and interquartile range (in brackets). Categorical variables (sex, and asthma control) were summarized as counts for each group and percentages (in brackets). Statistically significant *p*- values are in boldface (*p* -value < 0.05). Abbreviations: BMI: Body Mass Index; FVE_1: Forced Expiratory Volume; FVC: Forced Vital Capacity; IgE: Immunoglobulin E; AAT: Alpha 1-antitrypsin; n: sample size.

*Pi*S* and *Pi*Z* alleles of *SERPINA1* gene are associated with specific variants of a BRD4-Independent Enhancer

Ainhoa Escuela-Escobar, Esther Herrera-Luis, Elena Martín-González, José María Hernández-Pérez, Mario A. González Carracedo, and José Antonio Pérez Pérez

Table Supplementary 4. Clinical and demographic characteristics of studied individuals, considering *SERPINA1* genotypes.

Characteristics	Asthmatic patients					Newborns				
	MM (n = 335)	MS (n = 70)	*p*- value	MZ (n = 28)	*p*- value	MM (n = 375)	MS (n = 68)	*p*- value	MZ (n = 16)	*p*- value
**Age (years)**	47.1 (32.0 - 63.0)	47.0 (30.5 - 66.0)	0.953	51.6 (41.3 - 62.5)	0.233	NA	NA	NA	NA	NA
**Sex (female), n (%)**	215 (64.2)	46 (65.7)	0.915	18 (64.3)	1.000	169 (45.1)	37 (54.4)	0.204	8 (50.0)	0.903
**BMI**	28.5 (24.0 - 32.4)	28.3 (23.6 - 32.0)	0.806	30.4 (27.4 - 33.9)	0.079	NA	NA	NA	NA	NA
**FEV_1/FVC (% predicted)**	0.8 (0.7 - 0.9)	0.8 (0.7 - 0.9)	0.483	0.8 (0.8 - 0.9)	0.172	NA	NA	NA	NA	NA
**IgE levels (UI/ml)**	148.4 (31.7 - 229.6)	156.7 (40.9 - 222.3)	0.677	164.2 (34.5 - 244.0)	0.723	NA	NA	NA	NA	NA
**Eosinophil counts (cells/µl)**	280.7 (200.0 - 400.0)	280.6 (100.0 - 300.0)	0.583	297.7 (200.0 - 400.0)	0.586	NA	NA	NA	NA	NA
**AAT levels (mg/dl)**	131.1 (117.1 - 141.0)	116.1 (101.4 - 125.9)	**$6.68x10^{-09}$**	81.9 (76.8 - 87.7)	**$2.20x10^{-16}$**	NA	NA	NA	NA	NA
**Asthma control, n (%)**	149 (44.5)	26 (31.1)	0.310	16 (57.1)	0.280	NA	NA	NA	NA	NA

Continuous variables (age, BMI, FVE_1/FVC, IgE levels, eosinophil counts, and AAT levels) were summarized with the median and interquartile range (in brackets). Categorical variables (sex, and asthma control) were summarized as counts for each group and percentages (in brackets). Statistically significant differences (Chi-squared test) respect to *Pi*MM* individuals are shown in boldface (*p* -value < 0.05). Abbreviations: BMI: Body Mass Index; FVE_1: Forced Expiratory Volume; FVC: Forced Vital Capacity; IgE: Immunoglobulin E; AAT: Alpha 1-antitrypsin; n: sample size.
